# On the Structure and Development of the Vertebrate Skeleton

**Published:** 1863-12

**Authors:** 


### ON THE STRUCTURE AND DEVELOPMENT OF THE VERTEBRATE SKELETON.

By Professor HUXLEY, F. R. S.

In the course of the preceding ten lectures I have done my best to confine myself to matters of fact, and to the interpretation which directly arises from a very simple method as applied to those facts. The matters of fact have, as far as practicable, been demonstrated before you; at any rate, they are capable of demonstration. The interpretation has rested upon a principle so obvious that it requires no special justification—the principle that those parts in various skulls are identical, and should receive the same names, which correspond in development, and in their relations to the soft parts and to one another. And for myself I confess that with the establishment of those matters of fact, of those direct interpretations, the great object of science seems to me to be largely fulfilled. But in the present condition of anatomical views upon the skull, a course of lectures upon the vertebrate cranium which should be devoid of some commentary upon theories which have been offered of the composition of the cranium would, justly enough, be considered imperfect; because there is a true theory as apart from the speculative hypotheses—there is a true theory, which is simply a generalized statement of the facts which we have found out; and there are other theories, which add to such generalized statement other considerations, and which may or may not be capable of verification.

That which is commonly understood now by the theory of the skull, is the doctrine that the osseous cranium—it must be borne in mind that the doctrine has gone no further than the osseous cranium—is made up of a greater or smaller number of coalesced vertebræ, and that, to use the accepted phrase, the skull is a modification of the vertebral column. That doctrine originated with a man of vast genius—a man, perhaps, unexampled in the history of letters and of science, as, combining as I think, the highest proficiency in both departments—I mean Gœthe. He originated, or rather I should say he invented, towards the end of the last century, the doctrine that the skull is composed of a number of segments, corresponding in their nature with the vertebræ. I am perfectly well aware that this has been controverted. I am well aware that, not only in his own country, where there were personal interests and prejudices coming into play, but even in this country, where no such motives could intervene, the claims of Gœthe to this discovery have been disputed; and not only that, but that it has been more than insinuated—and it is with regret I state it—that the grand old poet, at seventy years of age, full of all the honors which his people had conferred upon him, was guilty of the meanness of taking to himself the credit of the doctrine invented by a young and rising professor, ignoring his claims and setting up others which had no foundation. Nay, more; the audacity of the accuser has gone so far as to suggest a certain stupidity, and that even Gœthe—this man of vast imagination and undoubted ability—was constrained to invent the same method of accounting for his discovery as that which had been given by Oken. The statement has passed into English literature; it is to be found in a work which will be consulted by all who wish to be acquainted with this matter, and it will doubtless be regarded as authority—it is to be found in an article upon Oken in the "Encyclopœdia Britannica."

What, then, are the facts of the case? Gœthe, writing in the year 1820, or thereabouts, has stated that the mode of his discovery of the vertebral composition of the skull was this: —"The three hindermost parts," says he, "I knew before; but it was only in 1791, when I picked up an old and broken sheep's skull amidst the sandy dunes of the Jewish cemetery in Venice, that I perceived that the facial bones were also composed of vertebræ; and perceiving, as I did, the transition from the first pterygoid bone to the ethmoid bone and to the turbinals quite distinctly, there I had an *aperçu* of the whole at once." We shall see that Oken, writing in 1807, also said and no doubt with perfect truth, that he had been led to his conception of the theory of the vertebrate skull by picking up an old and whitened deer's skull upon the Hercynian, within the Hartz mountains; and it has been more than suggested that this statement of Gœthe's was a sort of clumsy fabrication imitating the invention of Oken. Happily, the general order of things in this world is tolerably just, and a document which Gœthe doubtless had himself utterly forgotten (and which it is pretty clear, by a comparison of dates, he had forgotten) has appeared within the last year or two, which places the veracity of the old poet beyond the reach of the most vigorous and determined assailant. That passage was brought to light by an eminent German author a year or two ago, who says (referring to a correspondence which had just then been recently published between Gœthe and the family of Herder, the clergyman and great literary German) Gœthe was in the habit of corresponding with these people regularly; and he wrote, among other folks, to Madame Herder. One of his letters has come to light under the date of the 4th of May, 1790, and in that letter is this passage:—"By the oddest happy chance, my servant, by way of a joke, picked up a bit of an animal's skull in the Jews' Cemetery at Venice, and, by way of making fun, offered it to me as if he were presenting me with a Jew's skull. I have made a great step in the explanation of the formation of animals." It is quite clear that this is a complete and perfect testimony to the veracity of Gœthe when, in consequence of various circumstances that took place, he affirmed that thirty years ago (it was exactly thirty years ago) he had made the discovery to which I here refer. It is to be hoped now that all further detraction, at any rate, will cease on that point. And this detraction was the more unwise because it was wholly unnecessary. No person looking at the history of the past would dream of regarding Gœthe, who indubitably invented this doctrine, but who kept it to himself for thirty years—led to that course doubtless by the great difficulties which his vast knowledge and clear judgment showed him were in the way of the application of the doctrine—I say, no one would have been led to set up his claim as a discoverer against that which is justly the right of Oken, who, in the year 1807, in a similar manner to that which happened to Gœthe, was led, by picking up an animal's skull in the Hartz mountains, as I have said, suddenly to conceive that the osseous cranium was composed of three segments, and that the three segments answered exactly and precisely to so many vertebræ. No doubt Oken was the first promulgator of this doctrine; to him is all the credit that may attach to it justly due—all the credit that may attach to the definite discovery of the segmentation of the skull. Whatever we may think about his vertebral theory, no one can doubt at all that the merit of the discovery of the osseous segmentation of the skull is indubitably due to Oken; nor has anything been done since which in any way exceeds in merit that first singular paper which he published on the subject in 1807, under the head of "Ueber die Bedeutung der Schadelknochen." It was his inaugural address on taking the chair at the University of Jena; and to show you how easily this notion may have arisen,

I have here that subject which led Gœthe to imagine the vertebral theory, and that subject which Oken made use of as the best illustration of it—I mean a young sheep's skull. The parts here have simply been macerated, and otherwise are left free in their natural connection; but you will see that it is perfectly easy, by exercising a little pressure, putting in the first place a knife between the supra occipital, to separate one segment between the supra occipital and the parietals, and, putting a knife again between the parietals and frontals, to separate another, and thus to obtain three perfectly distinct osseous segments. It was that which Oken saw in the bleached deer's skull; it was that which very naturally, and as I think very consistently, led him to say (he says the conviction came across him as a lightning flash) that this great skull, which contains the diluted continuation of the spinal marrow, is itself nothing more than a diluted continuation of the vertebral column. In his first essay he is guided entirely by the analogy of the sheep's cranium, and he says that the hindermost vertebra, which he calls the ear vertebra, is composed of the basilar portion of the occipital bones and the two ex-occipitals—the two auricular portions, and the supra-occipital; that the next segment is formed by the posterior sphenoid, by the great alæ of the sphenoid, and by the parietals; and that the anterior segment is formed by the pre-sphenoid, by the little orbito-sphenoids, and by the frontals. Use the term segments, leaving the doubtful term vertebræ out of place, and there cannot be a doubt that Oken was perfectly justified, and that the fact will remain as he stated it to be for all time, as one which you can always demonstrate in the osseous skull. Then he goes on to say that there is an anterior portion, consisting of the ethmoid, the nasals, and the vomer, and that you can take that away. He is somewhat doubtful as to what is the nature of this; but, at any rate, he suggests as a possibility that the vomer may be in reality the continuation forward of the vertebral axis formed by the pre-sphenoid, the basi-sphenoid, and the basi-occipital; and that perhaps it may represent two or three vertebræ coalesced. He appears to be misled by some statement on this subject by an anatomical writer; but that point he leaves open. At first he stands simply by the three cranial vertebræ, as he calls them. He says: "You may think, perhaps, that I have forgotten the petrosal bone. Not at all. This petrosal bone is not a vertebra; it contains no parts of one of its vertebral components of the skull; but it is a sensory organ; it is an ossification developed around the organ of hearing which characterizes the formation of that organ, in just the same way as the membranous case around the eye is peculiar to that organ. Therefore you must regard this as something apart from the segments of the vertebræ, and look upon it as a sense capsule—not considering it as entering into the composition of the skull at all." Having arrived at this notion of the resemblance of the upper arches of the skull to the cavity of the upper arches of the vertebræ in the trunk the next step was a very simple and very obvious one. Finding in the skull, so far, a perfect resemblance to the vertebral column, Oken, not unnaturally, arrived at the conclusion that the lower arches of the skull—the parts of the face—must answer in some way or other to the parts of the trunk; and carrying out that idea with the boldness which was characteristic of him—indeed, I might almost have said with the rashness which certainly characterized the majority of his later speculations—he said: "If I find the repetition of this upper part of the vertebral column in the skull, I must also find a repetition of the lower part; and as I find there a thorax and abdomen, so I must find a thorax and abdomen in the skull"; and he said that the thorax of the skull was formed by the palatine bones and the adjoining parts, and that the abdomen of the skull was formed by the oral cavity. The idea is not so far-fetched as you might at first imagine. There is the same sort of *à priori* probability about it that there is about the identification of the upper arches of the skull with the upper arches of the vertebral column. By this time the notion of identity of composition as he progressed appears to have taken full possession of his mind, and he said to himself, "If we can find the upper and lower arches of the trunk in the head, we ought to be able to find the limbs." And totally undeterred by the difficulties which would have suggested themselves to a man of less daring in this matter—and those difficulties which, as I believe, kept Gœthe from promulgating his doctrine—he said: "I will find all these parts. There is the shoulder-blade of the head in the squamosal bone; there is the arm of the head in the jugal apparatus; and there is the hand of the head in the upper maxilla; and there are the fingers of the head in the teeth; and there is the thumb of the head in the pre-maxilla." If you had asked Oken how he knew that these were the fingers and thumbs, and so forth, he would probably have been puzzled for an answer. He would have told you that the idea dominated over all these things, and that that great perception of an archetype, which was perceived perhaps only by the man of genius, could not be seen by inferior people who were simply looking at the facts; and he said, therefore, that the arm of the head was attached to the side of the head, fixed to it, and that the two hands of the head were, so to speak, expanded to the sides of the nasal capsule. Where, then, were the legs of the head? Nothing daunted, Oken said that they were to be found in the lower jaw, and that the teeth of the lower jaw were the toes of the head in the same way, and that the hyoidean apparatus was nothing more nor less than the pelvis of the head. One would have thought that the difficulty of finding two legs of the head attached to scapulæ of the head here was rather a strong one; but Oken has an answer for all these matters. It is not worth while to enter into them largely now, having other things to attend to. It does not seem to have entered his mind that for anything like scientific speculation you must have a criterion of the truth or falsehood of what you say, and that unless you have some such criterion, you can go on inventing theories and counter-theories until Doomsday, every one of which shall be just as good as every other. A few years afterwards, Oken enlarged his idea by regarding the nasal apparatus as a fourth vertebra, and thus arose that view of the skull as composed of four coalesced vertebræ, which has formed, without any considerable or material alteration, the basis of every speculation that has been published since. In this way Oken founded a school. His ideas were received with open arms in Germany, and his contemporaries, some of them, carried to a wonderful pitch the wildnesses—I had almost used a stronger word—with which the ingenious speculations of Oken are, to a vast extent, mixed. These, like Spix, for example, propounded the most extraordinary notions of the composition of the skull—notions which one must acquaint oneself with as a matter of history; but when one has done that, the best thing to do is to forget them, and to think of them no more. Others, more judicious, more accurate in thought, like Bojanus, enlarged the doctrine in other directions; but altered nothing, added nothing whatsoever to the method of Oken, but simply shifted backwards and forwards the lines of interpretation which he had suggested. By degrees the notion spread in France, or rather I should say that it had an independent origin there to a certain extent, partly depending upon an interchange of ideas with Germany, partly arising as the result of the development of notions which Geoffrey St. Hilaire propounded in France upon the unity of organization, and so forth. Thus further modifications were produced, some admitting three vertebræ, some making out that there were four, some that there were six, and some that there were seven; but the character of the reasoning and the method of the interpretation were not one whit changed from that which was invented by Oken.

In England, again, we have had our own propounders of similar theories. I do not know that anything which I have said about Geoffrey St. Hilaire and about the Germans does not equally apply to them. I am quite at a loss to find in our English speculations any advance whatsoever upon the method, or in the main upon the facts, of Oken. If it is absurd, without good evidence, to talk of the jugal apparatus and the squamosal apparatus, and the upper jaw, as being the arm of the head, it is at least as absurd, without equally good evidence, and upon mere fantastical grounds, to regard the arm as a rib of the head. The one notion has exactly the same value and scientific standing as the other, and if the transpositions of Oken shock the mind, not less do those who are accustomed to the study and careful interpretations which result from embryology feel astounded in having to believe that the appendage of the atlas has, somehow or other, got in front of the rib of the occipital bone, and thus given rise to the position of the parts as we find them. My business, however, is not to enter into a criticism of these theories, but simply to show what is their scientific position.

In France, however, and in England, there have been great exceptions to these mere blind developments of the notions of Oken. In France the enormous knowledge and accurate thought of Cuvier kept him from drifting into such conceptions. He admitted, as all must admit, that there was a segmentation of the skull; but the moment these notions of the vertebration of the skull were proposed, he said, "I want evidence; I want a criterion. It is of no use talking to me about the relative repetitions, shifting backwards and forwards and all the rest. I want something which shall prove to me that these processes really do take place, or I will not accept your interpretation." Therefore Cuvier always held aloof from the vertebral doctrine, and I confess for myself I fully sympathize with those occasionally somewhat bitter sarcasms with which he overwhelmed the advocates of those notions, pernicious to everything like accuracy of thought, in his "Ossemens Fossiles," and his "Histoire Naturelle des Poissons." In this country, again, there has been another exception. I allude to Professor Goodsir, of Edinburgh, and the able young men who have risen from his training. He is the only man, so far as I know, either on the Continent or here, who has understood the value of that which took place between 1837 and 1840, or thereabouts, in Germany (of which I shall have to speak presently), and has endeavored to correct the errors of the merely Okenian line of speculation by the severe criteria of embryology. What was it, then, that took place between those years, 1837 to 1840, in Germany. It was a revolution in method—it was the discovery of that criterion for the want of which this wild and waste method of speculation upon the nature of the skull had originated. At about the period to which we refer, Reichert and Rathke, two men whose names must always be mentioned with the profoundest respect in connection with the skull, commenced their embryological researches, and instead of confining themselves to the easy task of sitting in their chairs and speculating backwards and forwards as to how the bones of the skull should be fitted together, like a child's puzzle, they set to work to ascertain how they had really come, and by the most laborious and difficult studies of development, to see in what manner the complex adult skull had arisen from its early state, and how, by tracing back the methods of development, by tracing back every skull to that early condition in which it resembles every other skull, you are able to identify the precise places at which each bone arose, and so to eliminate from your mind the mystifying influences of subsequent change. These men—Reichert, by his discovery of the visceral arches, and by his wonderful study of their modification; Rathke, by his investigations in comparative embryology, and his still more remarkable discoveries, as I think, into the true nature of the base of the skull, and the mode of its production—these men founded an absolutely new epoch and a new method; and for me any work which has been published since that time, and which contains no reference to the labors of these men, and ignores them, is on exactly the same scientific footing as a treatise on astronomy written at the present day which should ignore the discoveries of Newton; it is an anachronism, has no scientific place, and is unworthy of consideration.—*London Lancet.*

